# Isolation and Characterization of a Lycopene ε-Cyclase Gene of *Chlorella *(*Chromochloris*) *zofingiensis*. Regulation of the Carotenogenic Pathway by Nitrogen and Light

**DOI:** 10.3390/md10092069

**Published:** 2012-09-21

**Authors:** Baldo F. Cordero, Inmaculada Couso, Rosa Leon, Herminia Rodriguez, Maria Angeles Vargas

**Affiliations:** 1 Institute of Plant Biochemistry and Photosynthesis, CIC Cartuja, University of Seville and CSIC, Avda. Americo Vespucio, n° 49, Seville 41092, Spain; Email: baldomero@ibvf.csic.es (B.F.C.); inmaculada.couso@ibvf.csic.es (I.C.); avargas@us.es (M.A.V.); 2 Department of Chemistry, Experimental Sciences Faculty, University of Huelva, Avda. Fuerzas Armadas s/n, Huelva 27071, Spain; Email: rleon@uhu.es

**Keywords:** carotenoids, microalgae, lycopene cyclase genes, gene expression, gene regulation

## Abstract

The isolation and characterization of the lycopene ε-cyclase gene from the green microalga *Chlorella *(*Chromochloris*) *zofingiensis* (*Czlcy-e*) was performed. This gene is involved in the formation of the carotenoids α-carotene and lutein. *Czlcy-e* gene encoded a polypeptide of 654 amino acids. A single copy of *Czlcy-e* was found in *C. zofingiensis*. Functional analysis by heterologous complementation in *Escherichia coli* showed the ability of this protein to convert lycopene to δ-carotene. In addition, the regulation of the carotenogenic pathway by light and nitrogen was also studied in *C. zofingiensis*. High irradiance stress did not increase mRNA levels of neither lycopene β*-*cyclase gene (*lcy-b*) nor lycopene ε-cyclase gene (*lcy-e*) as compared with low irradiance conditions, whereas the transcript levels of *psy*, *pds*, *chyB* and *bkt* genes were enhanced, nevertheless triggering the synthesis of the secondary carotenoids astaxanthin, canthaxanthin and zeaxanthin and decreasing the levels of the primary carotenoids α-carotene, lutein, violaxanthin and β-carotene. Nitrogen starvation *per se* enhanced mRNA levels of all genes considered, except *lcy-e and pds*, but did not trigger the synthesis of astaxanthin, canthaxanthin nor zeaxanthin. The combined effect of both high light and nitrogen starvation stresses enhanced significantly the accumulation of these carotenoids as well as the transcript levels of *bkt* gene, as compared with the effect of only high irradiance stress.

## 1. Introduction

**Figure 1:**
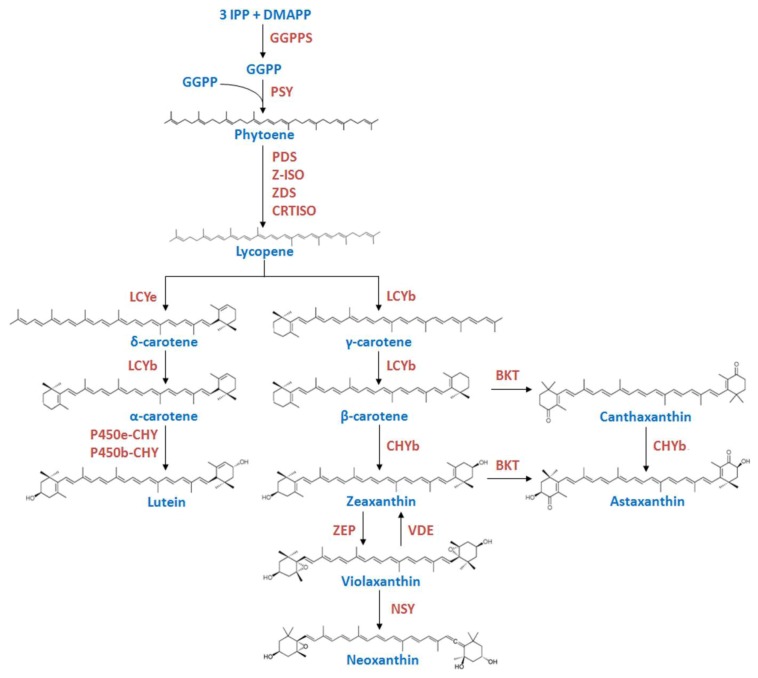
Schematic diagram of the carotenoid biosynthetic pathway in plants and green algae. IPP, isopentenyl pyrophosphate; DMAPP, dimethylallyl pyrophosphate; GGPP, geranylgeranyl pyrophosphate; GGPPS, geranylgeranyl pyrophosphate synthase; PSY, phytoene synthase; PDS, phytoene desaturase; Z-ISO, 15-*cis*-ζ-carotene isomerase; ZDS, ζ-carotene isomerase; CRTISO, carotene isomerase; LCYe, lycopene ε-cyclase; LCYb, lycopene β-cyclase; P450e-CHY, cytochrome P450 ε-hydroxylase; P450b-CHY, cytochrome P450 β-hydroxylase; CHYb, carotene β-hydroxylase; BKT, β-carotene oxygenase; ZEP, zeaxanthin epoxidase; VDE, violaxanthin de-epoxidase; NSY, neoxanthin synthase.

Carotenoids are essential pigments for all photosynthetic organisms as concerns participation in light harvesting, photoprotection, structural maintenance of pigment-protein complexes and membrane structure and fluidity [1,2,3]. In chloroplasts of plants and algae, the carotenoids precursor, geranylgeranyl pyrophosphate (GGPP), is synthesized by the action of the GGPP synthase from isopentenyl pyrophosphate and dimethylallyl pyrophosphate, which are derived from deoxyxylulose 5-phosphate pathway. The condensation of two GGPP molecules produces the first carotene, phytoene, catalyzed by phytoene synthase (PSY) (Figure 1).

PSY has been shown to be rate-limiting for carotenoid synthesis in plants [4,5] and algae [6]. Phytoene is desaturated by phytoene and ζ-carotene desaturases (PDS and ZDS) and isomerized by 15-*cis*-ζ-carotene isomerase (Z-ISO) [7] and carotene isomerase (CRTISO) to form the linear all *trans*-lycopene. Lycopene is converted into either β-carotene by the action of lycopene β-cyclase (LCYb) or into α-carotene by the action of lycopene ε-cyclase (LCYe) and LCYb. The cyclation of lycopene into either α- or β-carotene is a key branch point in the pathway of carotenoid biosynthesis in plants and some algal classes and has been proposed as a control step [8]; the relative activities of LCYe and LCYb determine the proportion of carotenoids directed to each branch of the pathway. α-carotene is modified into lutein by the hydroxylases P450b-CHY and P450e-CHY, and β-carotene is hydroxylated by CHYb to zeaxanthin. Zeaxanthin epoxidase (ZEP) and violaxanthin de-epoxidase (VDE) catalyze the interconversion of zeaxanthin and violaxanthin [5,9], and neoxanthin is formed from violaxanthin by the action of neoxanthin synthase (NSY). A limited number of organisms including some green algae such as *Haematococcus pluvialis* and *C. zofingiensis* can synthesize astaxanthin from β-carotene by the action of a ketolase/oxygenase (BKT) and the hydroxylase (CHYb) [10,11,12,13,14].

Currently, lutein and astaxanthin are widely used as feed additives in poultry farming and aquaculture. They have also important applications in food, nutraceutical and pharmaceutical industries because of their antioxidant activity and beneficial effects on human health [15,16]. 

*C. zofingiensis* is a model organism for the study of the regulation of the carotenoids biosynthetic pathway, since it produces both the primary carotenoids lutein and violaxanthin under standard growth conditions, as well as the secondary carotenoids astaxanthin, canthaxanthin and zeaxanthin under stress conditions such as high irradiance and nitrogen starvation or NaCl stress [17,18,19,20,21]. In recent years, some carotenogenic genes, namely *pds*, *bkt*, and *chyB* have been isolated and characterized in this microalga [12,13,22,23] and their regulation by light, NaCl, and different organic carbon sources has also been studied. High light stress up-regulated the transcripts of *pds*, *chyB* and *bkt* and NaCl stress only up-regulated the transcript levels of *bkt* [22,24]. The astaxanthin contents and the expression of *pds*, *chyB* and *bkt *genes were increased by glucose, sucrose or mannose addition to cells grown either at low irradiance [12,22] or in the dark [13,22].

Recently, we have isolated and characterized the carotenogenic genes *psy *and *lcy-b *from *C. zofingiensis* and have studied the effect of light and nitrogen on transcript levels of *lcy-b *gene, as well as on lutein and astaxanthin accumulation [6,25]. High irradiance stress did not up-regulate the transcripts of *lcy-b*, although it triggered astaxanthin synthesis. In contrast, nitrogen starvation increased mRNA levels of *lcy-b* but did not trigger the synthesis of astaxanthin. Nevertheless, the combination of high irradiance and nitrogen deprivation led to a significant enhancement of the astaxanthin accumulation accompanied by a decrease in lutein levels. 

In this paper, we report the isolation and characterization of the *lcy-e* gene from *C. zofingiensis*, a very unknown gene in algae, as well as confirm its ability to convert lycopene into δ-carotene. A general regulation study of the carotenogenic pathway, considering the new isolated *Czlcy-e* gene and other genes of the pathway in response to light and nitrogen has also been performed. 

## 2. Results

### 2.1. Isolation and Characterization of the *lcy-e* Gene and Deduced Protein from *C. zofingiensis*

**Figure 2:**
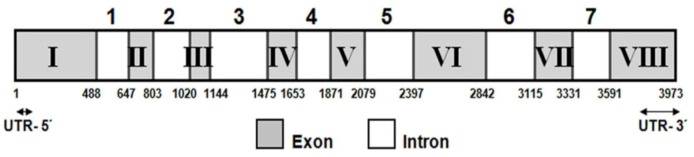
*Czlcy-e* gene organization. The diagram shows that the *Czlcy-e* gene consists of eight exons (I-VIII) and seven introns (1-7). The 5′ UTR and 3′ UTR sequences are indicated with arrows. Numbers indicate cDNA sequence coordinates (bp). UTR, unstranslated region.

Different pairs of degenerate primers were designed on the basis of the conserved motifs present in *lcy-e* from microalgae. A partial cDNA fragment of 669 bp was isolated by PCR amplification using degenerate primers (*lcy-e*-1F and *lcy-e*-1R) (Table 1). A complete BLAST homology searches in the Genbank database showed that this fragment had enough similarity with the *lcy-e* gene from other species and provided sequence information for designing specific primers for rapid amplification of 5′ and 3′ cDNA ends (RACE-PCR). This analysis generated a full-length cDNA of 2204 bp, which contained an ORF of 1965 bp, 2-nucleotides of 5′-untranslated region (UTR), and a long 3′ UTR of 237 nucleotides. A typical algal polyadenylation signal TGTAAA [26] was present in the 3′ UTR at 82 nucleotides upstream from the beginning of the poly(A) tail. The predicted protein has 654 amino acids residues, with an estimated molecular weight of 71.1 kDa, a theoretical isoelectric point of 8.45 and an instability index of 43.6 (data obtained with ProtParam program, [27]). The differences between the *C. zofingiensis lcy-e* gene and the cDNA sequence were compared and revealed the presence of 8 exons and 7 introns. Exon size ranged between 124 (exon III) and 488 bp (exon I), and intron size between 159 (intron 1) and 331 bp (intron 3). Approximately 56% (2.2 out of 3.9 kb) of the *Czlcy-e* gene corresponded to exon sequences. In all introns, the consensus GT donor and AG acceptor sequences at the 5′ and 3′ termini were found (Figure 2).

**Table 1:** Nucleotide sequences of primer pairs used for PCR amplification. F: forward; R: reverse. ^a ^*Sac*I and *Hind*III sites (lowercase letters underlined) were added for cloning the gene into the corresponding cut sites of pQE-80L vector.

Primer	Sequence (5′→3′)
**Partial *lcy-e *fragment**	
lcy-e-1F	AACCGCGTGTTCCTGGARGARACNTG
lcy-e-1R	TGGCACAGCAGCTCCATNCCRAA
**5′ and 3′ RACE**	
GSP-F	GGCATCAAAGTCACACGCATACACG
GSP-R	ACTGAACCCTGTGGCGGGATGCACC
NGSP-F	ACCCTCAGCAAACCAGCCTATTACAGCT
NGSP-R	CTTGAAAGGCAGTGCTGGCTTAGCTA
**Genomic DNA amplification**	
lcy-e-2F	ACATGGGGACACCAGCAGCAACTG
lcy-e-2R	GCGAGGGGGTTGTGACTGCATCT
lcy-e-3F	CCAGCAAGACAAGCTCGCAGCAATG
lcy-e-3R	TGCACAGATCCACGAGGTGCTGGC
lcy-e-4F	GTGTTACTTTGGTGAGGGCAATCAGGTC
lcy-e-4R	CCACAAGCCATCATTAGCATTCGGGTGG
lcy-e-5F	GTTCATGGATTACAGAAGGCACCACACAGG
lcy-e-5R	TGACTCCCTGACAATGCTTGCACCGC
lcy-e-6F	TGGTGCATCCTGCCACAGGGTTCA
lcy-e-6R	CACCAGTCATAGCTGATTCCTTACTGCTCC
lcy-e-7F	GATCCTGCTGGCAGATACCTAATCAGTC
lcy-e-7R	GCAACTCTTGGCTTAAAGCTAGGTGC
**PCR for probe preparation**	
Czlcy-e-S-F	CCAGCAAGACAAGCTCGCAGCAATG
Czlcy-e-S-R	TGCACAGATCCACGAGGTGCTGGC
**Genetic complementation-pQE-80L ^a^**	
pQE-lcy-e-F	gagctcATGGGGACACCAGCAGCAACTGTA
pQE-lcy-e-R	aagcttTCACTGTTGCACCTGTGTTGCTGC
***Czlcy-e* expression**	
RT-Czlcy-e-F	TCAAAGCACAGGCGAACAAACA
RT-Czlcy-e-R	AACGTCGGGACCTATAAGTCCG
***Czlcy-b* expression**	
RT-Czlcy-b-F	CGCAGGCGAAAAATTCCTGTCA
RT-Czlcy-b-R	TAAGGAATGTCACACCGCTGGC
***Czpsy* expression**	
RT-Czpsy-F	CACCAGGTTGTCAGAGTCCA
RT-Czpsy-R	ACTAGTGTGTTGCTGACTCT
***Czpds* expression**	
RT-Czpds-F	GCCAGAAAAGATCCAATTTG
RT-Czpds-R	CATGCTTCTCCCGCAAGAAC
***CzchyB* expression**	
RT-CzchyB-F	ATTGGAGGAGTGTTTGGCATGGAG
RT-CzchyB-R	AGATATCGTTGGCCTCGAATGGTC
***Czbkt* expression**	
RT-Czbkt-F	GTGGTTTGGCAGGTTTATGT
RT-Czbkt-R	AGAACAATCGGAACGCACTG

**Figure 3:**
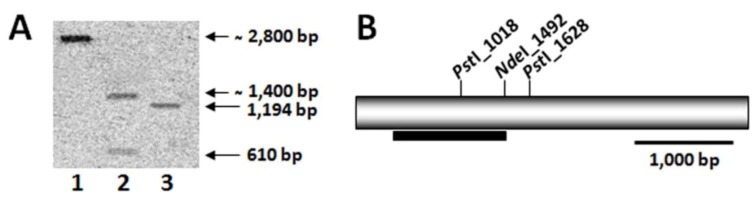
Southern blot analysis of genomic DNA from *C. zofingiensis*. (**A**) DNA was digested with *Nde*I (lane 1) or *Pst*I (lane 2), electrophoretically separated on a 0.8% agarose gel, blotted and hybridized at 65 °C with a probe of 1194 bp of the *lcy-e* gene amplified by PCR. A plasmid containing the *lcy-e* gene was used as a positive control (lane 3). (**B**) *Nde*I and *Pst*I restriction sites present in the *Czlcy-e* gene. The black bar indicates the probe location.

To determine the copy number of *lcy-e* gene in the genome of *C. zofingiensis*, genomic DNA was digested with two different restriction enzymes (either *Nde*I or *Pst*I) and subjected to Southern blot analysis at two different conditions of stringency, both in hibridization and washing (42 and 65 °C). Using a 1194-bp fragment of *Czlcy-e* as a probe, strong hybridization signals were obtained with both digestions. The digestion with *Pst*I enzyme, which cuts once inside the probe sequence, showed two bands, while digestion with *Nde*I, which cuts at one extreme of the probe, exhibited only one band for the two conditions of stringency tested (Figure 3). These results suggested the presence of a single copy of the *lcy-e* gene in the genome of *C. zofingiensis*.

**Figure 4:**
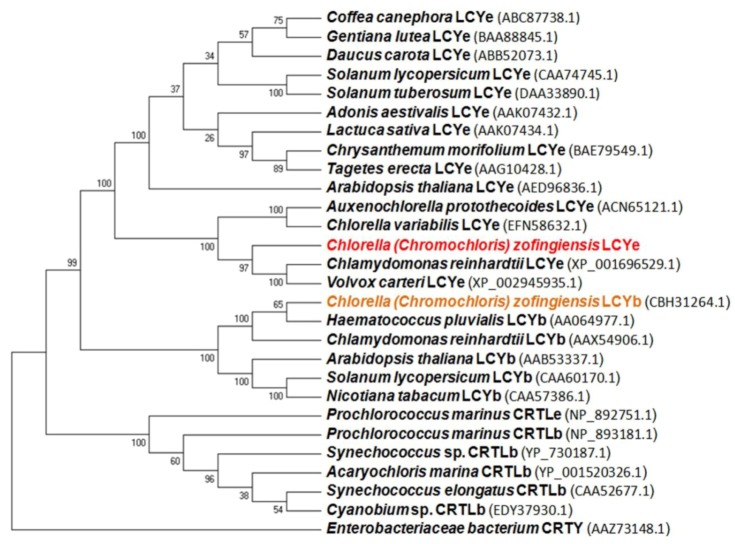
UPGMA tree analysis of the indicated plant, algal, cyanobacterial and bacterial lycopene cyclase amino acids sequences. Analysis was performed in MEGA5 [28]. The GenBank accession numbers for these species are shown. The tree is drawn to scale, with branch lengths in the same units as those of the evolutionary distances used to infer the phylogenetic tree. The evolutionary distances correspond to the number of amino acid substitutions per site and were computed using the Poisson correction method. *Numbers* at nodes indicate bootstrap values calculated over 500 replicates.

The BlastP search results demonstrated that the cloned CzLCYe showed the highest overall homology sequence with other LCYe from green algae, such as *Chlamydomonas reinhardtii *and *Volvox carteri *(identity 66%, similarity 77%) and *Auxenochlorella protothecoides *and *Chlorella variabilis* (identity 62%, similarity 74%). The GC content of the *Czlcy-e* coding region was 53%, which was lower than that of *C. reinhardtii *and *V. carteri *(63%) or of *A. protothecoides *(61%). The phylogenetic analysis of lycopene ε- and β-cyclases from green algae, cyanobacteria, plants and bacteria is illustrated in Figure 4. Analysis was conducted in MEGA5 using UPGMA method [28]. The predicted CzLCYe forms a cluster with the LCYe of green algae, which are phylogenetically close to LCYe of plants (~44% of identity, and 61% similarity). The degree of homology was lower with the LCYb of green algae, including *C. zofingiensis*, and plants (about 39% identity and 55% similarity) and with both cyanobacterial cyclases (CRTLe and CRTLb) (around 35% identity and 51% similarity). As other algal LCYe, CzLCYe was distantly related to bacterial CRTY cyclases, sharing with them only a few conserved motifs and about 23% identity. The characteristic Rossmann or dinucleotide binding fold and two cyclase motifs, present in LCYe and LCYb of green algae and plants, CRTLe and CRTLb of cyanobacteria and CRTY of bacteria, were also identified in the LCYe of *C. zofingiensis*, between amino acids 187 and 215, 405 and 421, and 480 and 489, respectively. In addition, a leucine in a region near the C-terminus, which was demonstrated to determine ε-monocyclase activity, was also identified in LCYe of *C. zofingiensis*. One basic amino acid histidine or lysine in that position was found in lycopene ε-cyclases from *Lactuca sativa* and *Prochlorococcus marinus* MED4 both of them showing ε-bicyclase activity [29,30]. 

Since microalgae and plants LCYe is located in the chloroplast membranes, we analyzed the CzLCYe sequence with different programs to determine both the presence of a signal peptide and transmembrane domains. The iPSORT program [31] predicted a putative plastid localization for the CzLCYe, and ChloroP 1.1 server [32] identified a chloroplast transit peptide at *N*-terminal end. Analysis with ProtScale [27] and TopPred [33] servers identified five deduced transmembrane domains of CzLCYe located between amino acids 1–21, 186–206, 472–492, 576–596 and 633–656 (data not shown). In addition, the predicted CzLCYe was highly hydrophobic. It had 46% of hydrophobic and non polar amino acids and 19% of charged amino acids. 

### 2.2. Functional Analysis of the CzLCYe in *E. coli*

**Figure 5:**
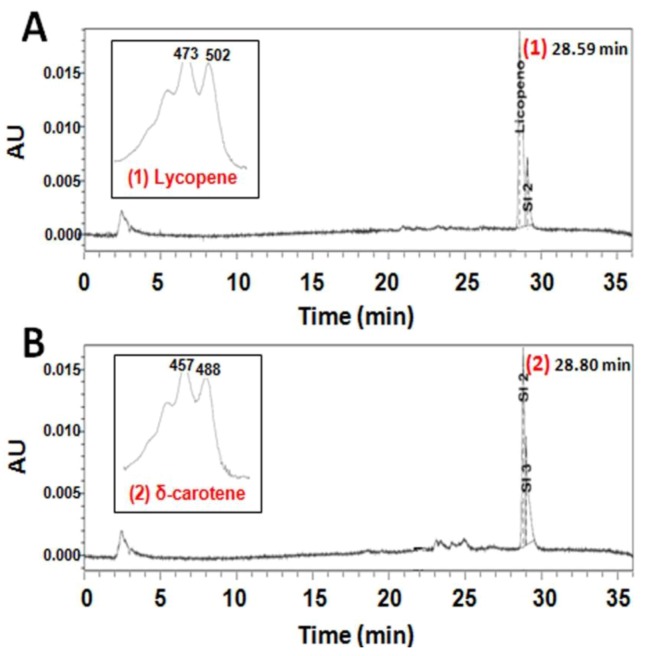
HPLC elution profiles of carotenoids extracted from cultures of *E. coli* carrying plasmids pAC-LYC (**A**) and pAC-LYC + pQE-*Czlcy-e* (**B**). The absorption spectra and retention time of the corresponding carotenoids are also shown. *E. coli* BL21 (DE3) cells transformed with the indicated plasmids were isolated in the presence of chloramphenicol (**A**) or chloramphenicol + ampicillin (**B**). Lycopene and δ-carotene were identified as described in Experimental Section. Peaks identification: (1) lycopene; (2) δ-carotene.

In order to check the functionality of the recently isolated gene, the full-length ORF of *Czlcy-e* was amplified and cloned into pQE-80L vector under the control of the β-galactosidase promoter. The resulting plasmid (pQE-*Czlcy-e*) was introduced in *E. coli* engineered to accumulate either lycopene or δ-carotene as final products, due to the presence of the plasmids pAC-LYC or pAC-DELTA, respectively. HPLC analysis of carotenoids extracted from *E. coli* showed that cells containing pAC-LYC produced lycopene (Figure 5A), while cells co-transformed with both pAC-LYC and pQE-*Czlcy-e* accumulated δ-carotene (Figure 5B). δ-carotene was also the only carotenoid synthesized by *E. coli* cells containing pAC-DELTA or both pAC-DELTA and pQE-*Czlcy-e* (data not shown). As negative controls, *E. coli* co-transformed with either pAC-LYC or pAC-DELTA and empty pQE-80L were used, resulting in the accumulation of lycopene or δ-carotene, respectively (data not shown). These results indicated that CzLCYe could catalyze the formation of one ε-ring at one of the ends of lineal lycopene to yield δ-carotene, but not the conversion of δ-carotene into ε-carotene (with one ε-ring in each end of lycopene), exhibiting, therefore, monocyclase activity. 

### 2.3. Expression of Carotenogenic Genes: Effect of Irradiance and Nitrogen

**Figure 6:**
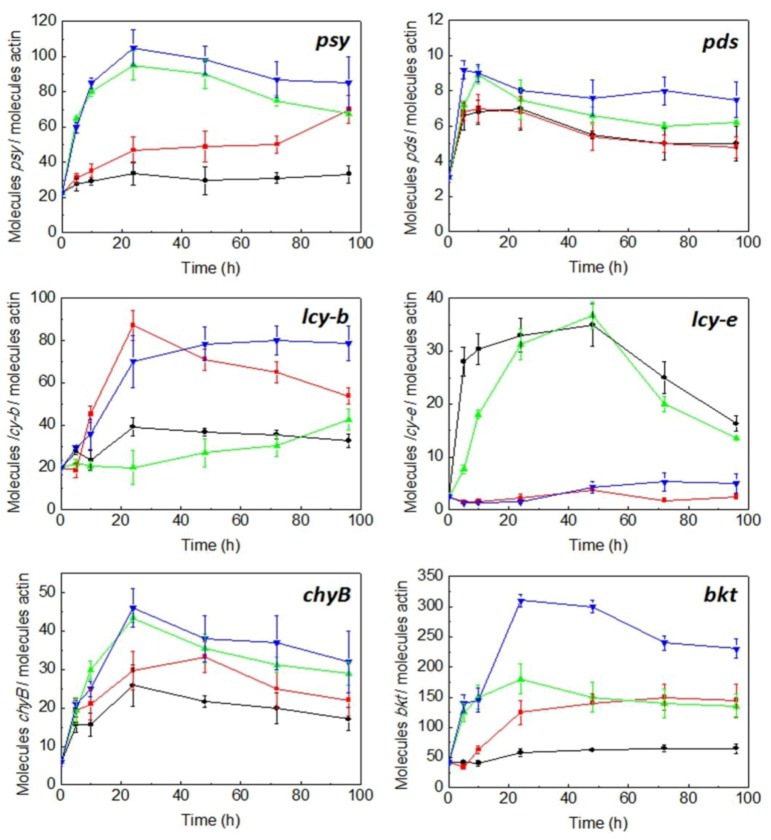
Effect of irradiance and nitrogen availability on the mRNA levels of the phytoene synthase (*psy*), phytoene desaturase (*pds*), lycopene β-cyclase (*lcy-b*), lycopene ε-cyclase (*lcy-e*), carotene β-hydroxylase (*chyB*) and β-carotene oxygenase (*bkt*) genes in *C. zofingiensis*. Culture conditions: low irradiance (20 µmol photons m^−2^ s^−1^) and nitrate replete (black line); low irradiance and nitrate deprivation (red line); high irradiance (300 µmol photons m^−2^ s^−1^) and nitrate replete (green line); high irradiance and nitrate deprivation (blue line). Error bars indicate the standard deviations of four independent measurements.

*C. zofingiensis* cells were grown photoautotrophically at low irradiance (20 μmol photon m^−2^ s^−1^) and a nitrogen replete concentration (nitrate 20 mM), as indicated in Experimental Section, until the middle of the exponential phase. Cells were then kept in the dark for 18 h, in order to make the mRNA levels come down to basal values. After this dark period, cells were subjected to either low (20 μmol photon m^−2^ s^−1^) or high irradiance (300 μmol photon m^−2^ s^−1^), under either nitrogen replete or nitrogen-deprivation conditions. Under conditions of nitrogen replete, nitrate concentration was measured daily (data not shown) and the nitrate consumed by the cells was added to the cultures. The evolution with time of transcriptional expression of the carotenogenic gene *lcy-e* as well as *psy*, *pds*, *lcy-b*, *chyB* and *bkt* as affected by irradiance and nitrogen availability was monitored by qRT-PCR. Changes in content of both the primary carotenoids lutein and α-carotene, β-carotene and violaxanthin and the secondary carotenoids astaxanthin, canthaxanthin and zeaxanthin were also determined in order to correlate transcript levels with the biosynthesis of them. As shown in Figure 6, under nitrogen replete concentrations and both at low and high irradiance, the relative transcript levels of *lcy-e* increased significantly, attaining 14-fold higher values than basal ones after 48 h, decreasing thereafter. On the contrary, nitrogen starvation did not affect the transcript levels of *lcy-e*, registering similar values to basal ones at both low and high irradiance. As previously described [25], the transcriptional expression of the *Czlcy-b* gene increased under nitrogen starvation (4-fold higher than basal levels), attaining a maximum after 24 h at low irradiance and decreasing later, similar maximal values being reached later, at 48 h, at high irradiance and being kept with time. Under nitrogen replete, no difference in maximal transcript levels either at high or low irradiance was observed. In the case of *psy*, mRNA levels at low irradiance and nitrogen replete were similar to those in the dark, increasing by about 5-fold when irradiance was raised from 20 to 300 µmol photons m^−2^ s^−1^, both under nitrogen starvation and nitrogen replete, attaining a peak after 24 h and decreasing slightly with time. At low irradiance, nitrogen starvation enhanced *psy* transcript levels by 3-fold with respect to the basal level after 96 h. The *pds* transcript levels, regardless of nitrogen availability, were two-fold higher at low irradiance after 24 h and three-fold higher at high irradiance after 5 h, when compared to basal levels, decreasing with time in both cases. The relative mRNA levels of *chyB* were about 4- and 7-fold higher than basal levels at low and high irradiance, respectively, under nitrogen replete, attaining a peak after 24 h. Nitrate deprivation increased slightly the transcript levels of *chyB* at low irradiance, showing similar values at high light intensity. In the case of *bkt*, mRNA levels at low irradiance and nitrogen replete were similar to basal values, increasing by about 4-fold when irradiance was raised from low to high light intensity, attaining a peak after 24 h and remaining constant with time. At low irradiance, nitrogen starvation enhanced *bkt* transcript levels by 3-fold with respect to the basal level after 24–48 h, remaining constant thereafter. The highest values were attained at high irradiance and nitrogen deprivation, reaching mRNA levels of *bkt* 7-fold higher as compared to the basal ones after 24 h, decreasing with time.

**Figure 7:**
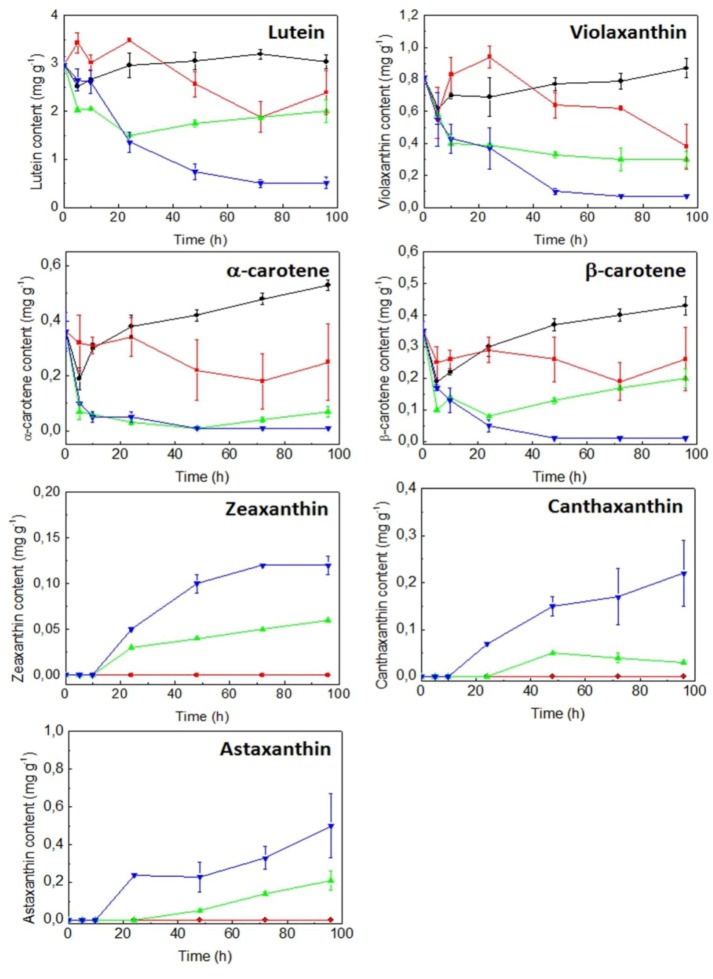
Effect of irradiance and nitrogen availability on the cellular content of lutein, violaxanthin, α-carotene, β-carotene, zeaxanthin, canthaxanthin and astaxanthin in *C. zofingiensis*. Culture conditions: low irradiance (20 µmol photons m^−2^ s^−1^) and nitrate replete (black line); low irradiance and nitrate deprivation (red line); high irradiance (300 µmol photons m^−2^ s^−1^) and nitrate replete (green line); high irradiance and nitrate deprivation (blue line). Carotenoids were identified as described in Experimental Section. Error bars indicate the standard deviations of four independent measurements. dw, dry weight.

With regard to cell primary carotenoid contents such as lutein, α-carotene, β-carotene and violaxanthin (Figure 7), they accumulated at low irradiance and enough nitrogen availability, cell contents decreasing at high irradiance and more significantly under conditions of both high irradiance and nitrogen starvation. An opposite trend was observed for secondary carotenoids, such as canthaxanthin, zeaxanthin and astaxanthin, their synthesis being triggered at high irradiance and the highest accumulations of these carotenoids being registered under conditions of nitrogen starvation. In addition, cells were lacking these carotenoids at low irradiance, regardless of the nitrogen availability. Total carotenoids and chlorophylls *a* and *b* contents showed a similar response than that registered for lutein under the different conditions studied (data not shown). 

## 3. Discussion

The carotenoid biosynthetic pathway is divided into two divergent branches at lycopene level in plants, some algae classes, such as green algae and certain cyanobacteria. In one branch, LCYb adds one β-ring at both ends of linear lycopene to form the β-β-carotenoid (β-carotene), which is transformed into zeaxanthin, violaxanthin and, only in some green algae, into astaxanthin (as *H. pluvialis* and *C. zofingiensis*). In the other branch, LCYe adds one ε-ring at one end of lycopene to form δ-carotene, which is transformed by LCYb into the β-ε-carotenoid, α-carotene, which is hydroxylated to lutein [3,29,34,35] (Figure 1). 

In algae, *lcy-e* gene has only been functionally characterized in the green alga *Auxenochlorella protothecoides* CS-41 [36] and, in cyanobacteria, in the divinyl-Chl *a*/*b*-containing cyanobacterium *Prochlorococcus marinus* MED4 [30]. We report here the isolation and characterization of the *lcy-e* gene isolated from *C. zofingiensis*. This new gene exhibited 8 exons and 7 introns, the ORF of the cDNA encoding a hypothetical protein of 654 amino acids (Figure 2). The length of this putative protein was slightly higher than those described for green algae and plants, being the cyanobacterial protein about 150 amino acids shorter, according to alignments performed with Blocks program (data not shown). This could indicate the existence of a signal peptide at the 5′ end of eukaryotic proteins. In fact, ChloroP program has confirmed the presence of a signal peptide in the CzLCYe. Although LCYe is an enzyme localized in chloroplast membranes, the predicted amino acid sequence of CzLCYe is not particularly hydrophobic. It has an average hydrophobic index of −0.078 and 19% of charged amino acids, and 46% are hydrophobic. However, by using Protscale and TopPred programs, five transmembrane domains of 20 amino acids each one uniformly distributed in the protein were found. A 42%–50% of hydrophobic amino acids were described for LCYe of plants and other green algae, as well as for other enzymes of the carotenogenic pathway, which were similar to that present in CzLCYe. However, the average hydrophobic index of these enzymes showed a higher variability, ranging from +0.116 for LCYe of *Volvox carteri* to −0.421 for PSY of *C. zofingiensis*. 

Four families of lycopene cyclases were identified: the monomeric lycopene β- and ε-cyclases from plants and some algae classes (LCYb and LCYe) and cyanobacteria (CRTLb and CRTLe); the usual monomeric bacterial lycopene β-cyclase (CRTY); the lycopene β-cyclases cruA and cruP, recently found in green sulphur bacteria and some cyanobacteria; and the heterodimeric lycopene β-cyclases (CRTYc and CRTYd) of some bacteria, which are related to the fungal bifunctional one (CRTYB) and include both PSY and lycopene β-cyclase activity. These families are distantly related to each other and share only a few conserved motifs, including a dinucleotide binding motif that is found in the first three groups but appears to be missing in the fourth [35,37,38,39]. This dinucleotide binding motif, as well as two cyclases motifs and a leucine residue in a region near the *C*-terminus indicative of ε-monocyclase activity were found in the predicted protein encoded by the new gene isolated from *C. zofingiensis* (data not shown). The predicted CzLCYe showed a high degree of homology with LCYe of plants, especially with the already known LCYe of other green algae, being the homology degree lower with the algae and plant LCYb and cyanobacterial CRTLe and CRTLb (Figure 4). These results would probably be enough to consider this new gene as a LCYe, but the functional analysis definitely confirmed this hypothesis. By complementation in *E. coli*, it was demonstrated that CzLCYe was able to catalyse the conversion of lycopene into δ-carotene, but not the formation of α-carotene from δ-carotene (Figure 5), exhibiting, therefore, monocyclase activity, as most LCYe of plant and algae previously studied. Two of the few examples of ε-cyclases that show bicyclase activity are a LCYe of romaine lettuce which adds two ε-rings to lycopene to form ε-carotene [29], and a CRTLe from *Prochlorococcus marinus* MED4 that adds not only ε- but also β-rings to lycopene to form α-, β- and ε-carotene [30]. In these two cyclases one basic amino acid histidine or lysine was identified in a region near the *C*-terminus instead of a leucine, which is found in all ε-monocyclases studied [29,30].

The cyclation of lycopene into either α- or β-carotene has been proposed as a control step in the carotenogenic pathway of plants. The relative activities of LCYe and LCYb may determine the flow of carbon through the carotenoids pathway from lycopene to either α- or β-carotene and their derivatives [29,34]. Indeed, by reducing the expression of *lcy-e* in *Arabidopsis*, *Brassica* and potato by mutation, either by using RNAi or by introducing an antisense fragment of this gene, the ratio of β- to α-carotene and their products increased [40,41,42]. A study of the natural carotenoid variation in maize uncovered alleles of *lcy-e* that are expressed at low levels also correlated with an increase in β-carotene and its derivatives [8]. On the other hand, overexpression of either the endogenous *lcy-b* gene or the equivalent heterologous genes in crop plants, such as *Brassica *seeds and tomato fruits increased β-carotene levels [43], and *lcy-e *overexpression in *Arabidopsis* increased lutein content up to 180% of wild type [44]. 

In photoautotrophically grown *C. zofingiensis*, it is known that the combination of both high irradiance and nitrogen starvation causes a drop in the cellular content in primary carotenoids, such as α-carotene and its derivative lutein, and a concomitant accumulation of secondary carotenoids, such as the β-carotene products astaxanthin and canthaxanthin [17,18,19]. However, the molecular basis of this regulation under those conditions is not yet well understood. Recently, it has been shown that high irradiance up-regulated the *pds*, *chyB and bkt *genes, enhancing significantly the synthesis of the β-carotene derivatives canthaxanthin, zeaxanthin and astaxanthin in this microalga [12,22,24], but did not affect the transcription of *lcy-b* gene [25]. On the other hand, nitrogen deprivation, regardless of irradiance, induced the transcription of *lcy-b* gene, however astaxanthin content increased at the expense of lutein under nitrogen deprivation but only at high irradiance [25]. In this work, we have investigated the regulation by irradiance and nitrogen of the carotenogenic pathway of *C. zofingiensis* by determining the mRNA levels of *lcy-b*, *psy*, *pds*, *chyB*, *bkt *in addition to the *lcy-e* gene, just now isolated by us, as well as the cellular contents in α-carotene and its product lutein and β-carotene and its derivatives canthaxanthin, astaxanthin, zeaxanthin and violaxanthin. According to our results, high irradiance stress did not increase mRNA levels of neither *lcy-b *nor *lcy-e* genes as compared to low irradiance conditions, whereas the transcript levels of *psy*, *pds*, *chyB* and *bkt* genes were enhanced significantly. However, high light stress triggered the synthesis of the secondary carotenoids astaxanthin, canthaxanthin and zeaxanthin and decreased the levels of the primary carotenoids α-carotene, lutein, β-carotene and violaxanthin. Therefore, in *C. zofingiensis*, high irradiance triggered the synthesis of astaxanthin, canthaxanthin and zeaxanthin by transcriptional up-regulation of the *psy*, *pds*, *chyB* and *bkt* genes, but not *lcy-b*, and, consequently, the regulation of this last gene must take place at a post-transcriptional level under the conditions mentioned above. In *H. pluvialis*, unlike in *C. zofingiensis*, an up-regulation of *lcy-b *by high light was shown astaxanthin synthesis also being induced under this high irradiance condition [45,46]. In *Dunaliella salina*, high irradiance stress increased slightly mRNA levels of *lcy-b*, *psy* and *pds* genes as well as the cellular β-carotene content, and the highest levels of these genes transcripts and β-carotene were obtained under high light combined with nutrient depletion. Nutrient limitation seems to be essential for β-carotene accumulation in this microalga [3,47,48].

In *C. zofingiensis*, nitrogen starvation *per se* enhanced mRNA levels of all genes considered, except *lcy-e and pds*, but did not trigger the synthesis of canthaxanthin, zeaxanthin nor astaxanthin. Nevertheless, the combination of both factors, high irradiance and nitrogen starvation, increased the levels of those carotenoids significantly. Therefore, the up-regulation of *lcy-b* but not of *lcy-e* by nitrogen starvation, regardless of irradiance, addressed the carbon flow from lycopene to β-carotene, canthaxanthin, astaxanthin and zeaxanthin instead of to α-carotene and lutein. However, nitrogen starvation under low irradiance did not trigger canthaxanthin, zeaxanthin and astaxanthin synthesis, although all up-stream and down-stream genes related to that branch of the pathway (except *pds*) were up-regulated, indicating either that the synthesis of all these carotenoids under the conditions mentioned above should be controlled at a post-transcriptional level or that *pds* is the main regulatory gene. The combined effect of both high light and nitrogen starvation stresses enhanced significantly the synthesis of canthaxanthin, zeaxanthin and astaxanthin, possibly due to a higher expression of *bkt*; on the contrary, lutein, α-carotene, β-carotene and violaxanthin decreased under these conditions. The decrease of violaxanthin content at high irradiance, especially under nitrogen deprivation, could be due to the increase in the accumulation of zeaxanthin through the xanthophylls cycle [49]. Therefore, although changes in the mRNA levels have been shown to be the most important regulation for carotenoid genes [3], post-transcriptional and translational levels also playing important roles, as well as the stability of RNA, as has been shown for *H. pluvialis *[3,45,46].

## 4. Experimental Section

### 4.1. Strains and Culture Conditions

The green microalga strain *Chlorella zofingiensis* SAG 211-14 (recently classified as *Chromochloris zofingiensis*, [50]) was obtained from the Culture Collection of Göttingen University (SAG, Germany). This microalga was maintained and grown photoautotrophically in Arnon medium [51] modified to contain 4 mM K_2_HPO_4_ and 20 mM NaNO_3_, at 25 °C under continuous illumination (50 µmol photons m^−2^ s^−1^), except where indicated. The liquid cultures were continuously bubbled with air, supplemented with 1% (v/v) CO_2_ as the only source of carbon. For the expression experiments, cells were grown in Roux flaks of 1 L capacity laterally and continuously illuminated with mercury halide lamps at either 20 (low irradiance) or 300 µmol photons m^−2^ s^−1^ (high irradiance) either in the presence or in the absence of nitrate. To keep constant the nitrate in the medium, the nitrate concentration was measured daily by HPLC according to Cordero *et al.* [25], and the nitrate consumed by the cells was added to the cultures. The light intensity was measured at the surface of the flasks using a LI-COR quantum sensor (model L1-1905B, Li-Cor, Inc. Lincoln, NE, USA).

*Escherichia coli *DH5α and BL21 strains were used as the hosts for DNA manipulation and for heterologous expression of *lcy-e* gene, respectively.

### 4.2. Genomic DNA and RNA Isolation and cDNA Preparation

DNA and total RNA were isolated using DNeasy Plant Mini Kit and RNeasy Plant Mini Kit (Qiagen, Düsseldorf, Germany), respectively. For Quantitative Real-Time PCR analysis (qRT-PCR), first-strand cDNA synthesis was obtained from total RNA treated with DNase as recommended by the manufacturer, by using the SuperScript First-Strand Synthesis System (Invitrogen, Barcelona, Spain) primed with oligo(dT)_18_ according to the manufacturer’s instructions.

### 4.3. Cloning of *C. zofingiensis lcy-e* cDNA and Genomic Gene

For isolating the* lcy-e* cDNA from *C. zofingiensis*, degenerate primers were designed from the conserved regions of LCYe from different species of algae. The PCR product was cloned in the pGEM-T vector (Promega, Madison, WI, USA) according to the manufacturer’s manual and then sequenced. The cDNA fragment obtained corresponding to partial *lcy-e* clone provided sequence information for the designing of gene-specific primers for amplification of 5′ and 3′ cDNA ends by RACE-PCR. All reactions were performed with kits according to the manufacturer’s instructions (Smart RACE cDNA Amplification Kit, Clontech, Mountain View, CA, USA). 5′ and 3′ RACE products were cloned into pGEM-T vector and sequenced. Specific primers were synthesized for genomic DNA amplification based on cDNA sequence. The primers sets used in this study are listed in Table 1.

### 4.4. Nucleotide Sequence Accession Numbers

The *Czlcy-e* cDNA and genomic DNA sequences have been registered in the EMBL database under the accession numbers HE664109 and HE664108, respectively.

### 4.5. Southern Blot Analysis

Genomic DNA was digested with *Nde*I and *Pst*I, which showed one recognition site in the probed region of the *lcy-e* gene. The probe was prepared by amplifying genomic DNA with the primers Czlcy-e-S-F and Czlcy-e-S-R, resulting in a 1194-bp fragment of *Czlcy-e* gene. The digested DNA was transferred to a Hybond-N membrane (GE Healthcare, Little Chanfont, UK) by capillary transfer and hybridized with the ^32^P labelled DNA probe at both low and high stringency overnight. After hybridization, the radioactivity of the membrane was monitored by the Cyclone Phosphor System (Perkin Elmer, Waltham, MA, USA). 

### 4.6. Functional Analysis of *Czlcy-e* cDNA by Heterologous Expression in *E. coli*

The *Czlcy-e* ORF was amplified by PCR with the primers pQE-lcy-e-F and pQE-lcy-e-R, which were designed to contain *Sac*I and *Hind*III restriction sites, respectively, and cloned into pQE-80L expression vector (Qiagen) resulting in plasmid pQE-*Czlcy-e*, which carries ampicillin resistance. The plasmids pAC-LYC and pAC-DELTA, kindly donated by Prof. Cunningham, carried the carotenoid pathway genes responsible for the synthesis of lycopene (*crtE*, *crtB*, and *crtI* of *Erwinia herbicola*) and δ-carotene (*crtE*, *crtB*, and *crtI* of *E. herbicola + lcy-e *of* Arabidopsis*), respectively [52,53]. Transformation of *E. coli* BL21 (DE3) with pQE-*Czlcy-e* and/or one of the two plasmids pAC-LYC or pAC-DELTA (both carrying chloramphenicol resistance) was made by electroporation. Transformed cells were plated on Luria-Bertani (LB) [54], supplemented with 100 μg mL^-1^ ampicillin and/or 40 μg mL^−1^ chloramphenicol, and grown at 37 °C for 1 day. The inducer isopropyl-β-D-1-thiogalactopyranoside (IPTG) was added at a final concentration of 1 mM. 

### 4.7. Quantitative RT-PCR

The mRNA relative abundance of *psy*, *pds*, *lcy-b*, *lcy-e*, *chyB* and *bkt* genes of *C. zofingiensis* was examined by qRT-PCR on an IQ5 Real-Time PCR Detection System (BioRad, Hercules, CA, USA), according to Cordero *et al.* (2010) [25]. In each experiment, a series of standard dilutions containing a specific concentration of a PCR fragment or a cDNA template was amplified in 20 µL of reaction containing 1× SYBR Green PCR Master Mix (Quantimix Easy SYG kit, BioTools B&M Labs, Madrid, Spain) and corresponding primers (Table 1). After heating at 95 °C for 10 min, cycling parameters were: 40 cycles of 95 °C for 30 s, 60 °C for 30 s, and 72 °C for 30 s. Finally, the specificity of the qRT-PCR products was confirmed by performing a melting temperature analysis at temperatures ranging from 55 to 95 °C at 0.5 °C per min and also by electrophoresis on a 2% agarose gel. Data were captured as amplification plots. Transcription levels of the target genes were calculated from the threshold cycle by interpolation from the standard curve. To standardize the results, the relative abundance of actin gene was also determined and used as the internal standard [45,55]. All calculations and statistical analyses were performed as described in the IQ5 Optical System Software 1.0 (BioRad). The complete experiments (RNA isolation, cDNA synthesis followed with qRT-PCR) were independently repeated twice, and the data were averaged. 

### 4.8. Analytical Methods

#### 4.8.1. Cell Concentration and Dry Weight Determinations

Cell number was determined with a Neubauer hemocytometer. For dry weight measurements, aliquots (5 mL) of the cell culture were filtered through Whatman GF/C paper (Whatman plc, Kent, UK), washed three times, and dried at 80 °C for 24 h.

#### 4.8.2. Carotenoid Extraction and HPLC Analysis

Pigments were extracted with methanol according to Cordero *et al.* (2010) [25]. The samples were then saponified with ethyl ether and KOH 2% in methanol [20] and then centrifuged and analyzed by HPLC using a Waters Spherisorb ODS2 column (4.6 × 250 mm, 5 µm particle size) (Waters, Mildford, MA, USA). The chromatographic method described by Cordero *et al.* (2010) was used [25]. Pigments were eluted at a flow rate of 1.0 mL min^−1^ and were detected at 440 nm using a Waters 2996 photodiode-array detector. Identification of carotenoids was achieved by comparison of the individual characteristic absorption spectrum and the retention time with known standards. The retention times of carotenoid analysed were: violaxanthin, 13.26 min; astaxanthin, 15.54 min; lutein, 18.10 min; zeaxanthin, 18.51 min; canthaxanthin, 20.51 min; lycopene, 28.59 min; δ-carotene, 28.80 min; α-carotene, 29.24 min; β-carotene, 29.52 min. Quantification was performed using a calibration curve generated with commercially available carotenoids standards from Sigma-Aldrich (St. Louis, MO, USA) and DHI (Holsholm, Germany).

## 5. Conclusions

Our results on the characterization of the *Czlcy-e* gene and the regulation by light and nitrogen of lycopene cyclase genes and other carotenogenic genes such as *psy*, *pds*, *chyB* and *bkt* from *C. zofingiensis* could contribute to the understanding of the regulatory mechanisms of the biosynthesis of carotenoids at the molecular level, which can be helpful for the optimization of the physiological conditions for high carotenoids production by *C. zofingiensis *(specially the very rare and of high industrial interest astaxanthin), and for performing metabolic and genetic engineering in this microalga, when transformation methods are well established. 
